# Prevalence and genotypes of infectious salmon anaemia virus (ISAV) in returning wild Atlantic salmon (*Salmo salar* L.) in northern Norway

**DOI:** 10.1111/jfd.13021

**Published:** 2019-06-13

**Authors:** Abdullah Sami Madhun, Stig Mæhle, Vidar Wennevik, Egil Karlsbakk

**Affiliations:** ^1^ Institute of Marine Research Bergen Norway; ^2^ Department of Biological Sciences University of Bergen Bergen Norway

**Keywords:** Aquaculture, Atlantic salmon, fish disease, infectious salmon anaemia virus, ISAV

Infectious salmon anaemia (ISA) is a serious viral disease of Atlantic salmon (*Salmo salar* L*.*). The ISA virus (ISAV) occurs both as apparently avirulent ISAV‐HPR0 variants with full length of haemagglutinin–esterase (HE) gene including the highly polymorphic region (HPR) and as virulent ISAV‐HPRΔ variants with various HPR deletions (Mjaaland et al., 2002). In addition, an insertion or a Q266L substitution in the fusion protein in segment 5 is a prerequisite for virulence (Markussen et al., 2008).

Infections with ISAV‐HPR0 are widespread in salmon aquaculture in Norway, Faroe Islands, Chile and Scotland (Christiansen, Ostergaard, Snow, Dale, & Falk, 2011; Godoy et al., 2013; Lyngstad et al., 2012; McBeath, Bain, & Snow, 2009; Vanderstichel et al., 2015). There is increasing evidence that all virulent ISAV strains have evolved from ISAV‐HPR0 progenitors (Christiansen et al., 2017).

Based on sequence analyses, ISAV can be divided into two major genetic groups: European (EU) and North American (NA). The EU group has been further divided into four clades (genogroups): three European (EU‐1 to EU‐3) and a European‐like group from north‐eastern North America (EU‐NA) (Christiansen et al., 2011; Devold, Karlsen, & Nylund, 2006; Nylund et al., 2007).

Very few “wild‐type” ISAV strains have been sequenced, so the phylogenetic placement of ISA viruses from wild Atlantic salmon is largely unknown (Cunningham, Gregory, Black, Simpson, & Raynard, 2002). In the current study, we investigated prevalence and genotypes of ISAV infections in returning wild Atlantic salmon from northern Norway.

A total of 419 Atlantic salmon were caught in 2012 at six sites distributed in three counties (Figure 1): Finnmark (Sites A, B and C), Troms (Site D) and Nordland (Sites E and F) (Madhun et al., 2018). Detection of ISAV in gill samples was performed by PatoGen Analyse AS using real‐time RT‐PCR assay which is designed to target the HE gene and validated for detection of both HPRΔ and HPR0 variants (Lyngstad et al., 2012).

**Figure 1:**
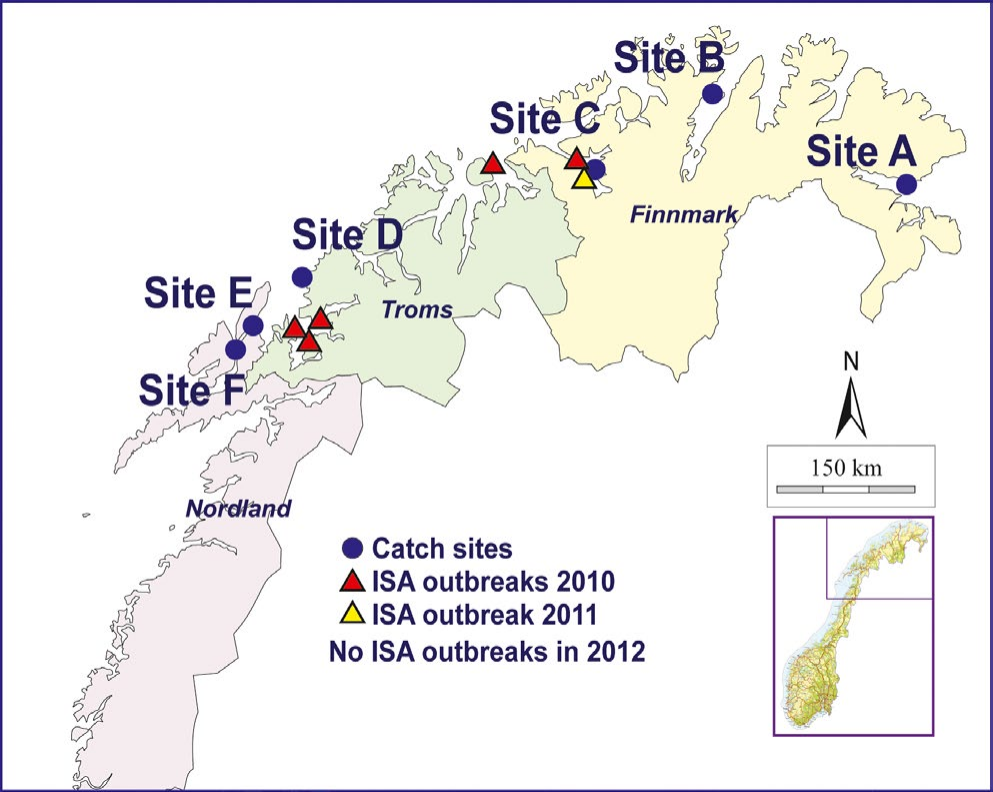
The location of salmon catching sites and the location of ISA outbreaks in fish farming between 2010 and 2011. No ISA outbreaks were recorded in 2012 [Colour figure can be viewed at http://www.wileyonlinelibrary.com]

Samples for sequencing of ISAV segments 5 and 6 were selected on the basis of *C*_t_ values (<35). The targeted gene sequences were amplified and sequenced as previously described (Kibenge et al., 2009; Vike, Nylund, & Nylund, 2009) using the primers shown in Table S1. The obtained sequences have GenBank accession numbers MH794610–MH794631. The HPR0 HE‐gene sequences obtained were aligned with all HPR0 HE‐gene sequences of similar length available in GenBank (per 12 January 2019). The complete alignment consisted of 78 sequences and 834 nucleotides.

Scale examination identified 42 (10%) salmon to be escapees from farms (Table 1). The wild salmon was dominated by 1‐ and 2‐sea‐winter (SW) fish. There were five, one and zero ISA outbreaks in the years 2010, 2011 and 2012, respectively (Figure 1). Therefore, 1‐SW and 2‐SW salmon from the outbreak areas may have been exposed to the virus during their migration to the ocean as post‐smolt. However, ISAV‐HPRΔ was not detected in any of the tested fish. On the other hand, ISAV‐HPR0 was detected in 6.9% of the captured salmon. The prevalence of ISAV‐HPR0 was 7.2% in returning wild salmon and 4.8% in escaped farmed fish (Table 1). However, there was no significant difference in the prevalence of ISAV‐HPR0 between the wild and the escaped farmed fish. This finding is interesting as previous reports have shown that escaped farmed fish are more frequently virus‐infected than wild salmon (Garseth, Biering, & Aunsmo, 2013; Madhun et al., 2015, 2017). This can be explained by the transient nature of ISAV‐HPR0 infection in salmon which may limit the detection‐time window of the virus (Christiansen et al., 2011).

**Table 1:** The origin and the percentage of ISAV‐HPR0‐infected salmon collected from different geographical sites in northern Norway

County Area	Number and origin of salmon			Number of ISAV‐HPR0‐positive salmon	
	Total	Wild	Escaped (% total)	Wild (% of wild)	Escaped (% of escaped)
Finnmark					
Site A	167	165	2 (1)	16 (9.7)	1 (50)
Site B	29	25	4 (14)	5 (20)	1 (25)
Site C	63	60	3 (5)	3 (5)	‐
Troms					
Site D	104	85	19 (18)	3 (3.4)	‐
Nordland					
Site E	34	25	9 (26)	‐	‐
Site F	22	17	5 (23)	‐	‐
Total	419	377	42 (10)	27 (7.2)	2 (4.8)

The prevalence of ISAV‐HPR0 infection in wild salmon varied between sites with the lowest prevalence in Sites E and F (0%) and the highest in Site A (9.7%) (Table 1). Consequently, the prevalence was highest in the areas with lowest fish‐farming intensities (Figure S1). Hence, the observed prevalence was not influenced by farming activities. It is likely that the ISAV‐HPR0 infections were recent, since ISAV‐HPR0 infections are not long‐lasting (Christiansen et al., 2011). Hence, the fish may have been infected at the oceanic feeding areas or in the coast. Studies investigating the occurrence of ISAV infections in wild salmon in the feeding areas are therefore needed. Our results highlight the potential role of wild salmon as a natural reservoir that may introduce ISAV‐HPR0 to farmed salmon (Christiansen et al., 2011; Nylund, Devold, Plarre, Isdal, & Aarseth, 2003).

Of the 29 ISAV‐HPR0‐positive fish, we were able to obtain 15 HE‐gene sequences. The phylogenetic relationship to other ISAV‐HPR0 sequences was examined (Figure 2). All our sequences grouped in a well‐supported clade together with HPR0 sequences from farmed salmon from Faroe Island (Christiansen et al., 2017) and northern Norway (Lyngstad et al., 2012; Plarre et al., 2012). This clade has previously been referred to as the EU‐G2 (Christiansen et al., 2017; Devold et al., 2006). Our sequences originate from 14 wild and one escaped farmed salmon caught in distantly separated sites in northern Norway (Figure 1). According to microsatellite‐based genetic stock identification and individual assignments of salmon to rivers (data not shown), three of the ISAV‐HPR0‐infected salmon likely originated from Kola Peninsula stocks (Pechenga and Kola rivers, Russia), while six others were from Finnmark County (mostly from River Alta) in Norway. Lyngstad et al. (2012) obtained 27 HPR0 sequences from farmed salmon collected mostly from middle and western Norway. However, 26 of these belonged to other genogroups (e.g., EU‐G1 and EU‐G3), while only a single HE‐gene sequence (FN687353; Figure 2), which was from northern Norway, grouped with the sequences reported in the current study. Unexpectedly, an ISAV‐HPR0 sequence from a Canadian farmed salmon in Newfoundland (Gagne & LeBlanc, 2018) also shows 100% identity to the present sequences from wild fish and others found in farmed salmon from the north‐east Atlantic. Gagne et al. (2018) suggested that wild salmon could be a potential source, since wild North American and European salmon intermingle in the oceanic feeding areas around the Faroes or West Greenland (Gilbey et al., 2017; Olafsson et al., 2016). The present observations support the existence of an ISAV‐HPR0 genogroup that is dominating in northern wild Atlantic salmon populations (Norway and Russia), which is also found in farmed salmon from the far north of Norway (Troms and Finnmark) and interestingly the Faroe Islands and east Canada. At present, the only report of ISAV‐HPR0 in wild salmon is from Scotland (Cunningham et al., 2002), showing a single HPR0 sequence which was identical to those obtained in the present study. This raises the question about how common this ISAV‐HPR0 genogroup is among wild salmon stocks throughout the North Atlantic. Another important question is whether infections with other ISAV‐HPR0 genogroups occur also among wild Atlantic salmon. Despite the expansion of salmon aquaculture and fish translocations within and across borders, ISAV among wild salmon stocks may still show a phylogeographical structure, which should be better known. Therefore, ISAV screening of wild Atlantic salmon from other geographical areas would be valuable.

**Figure 2:**
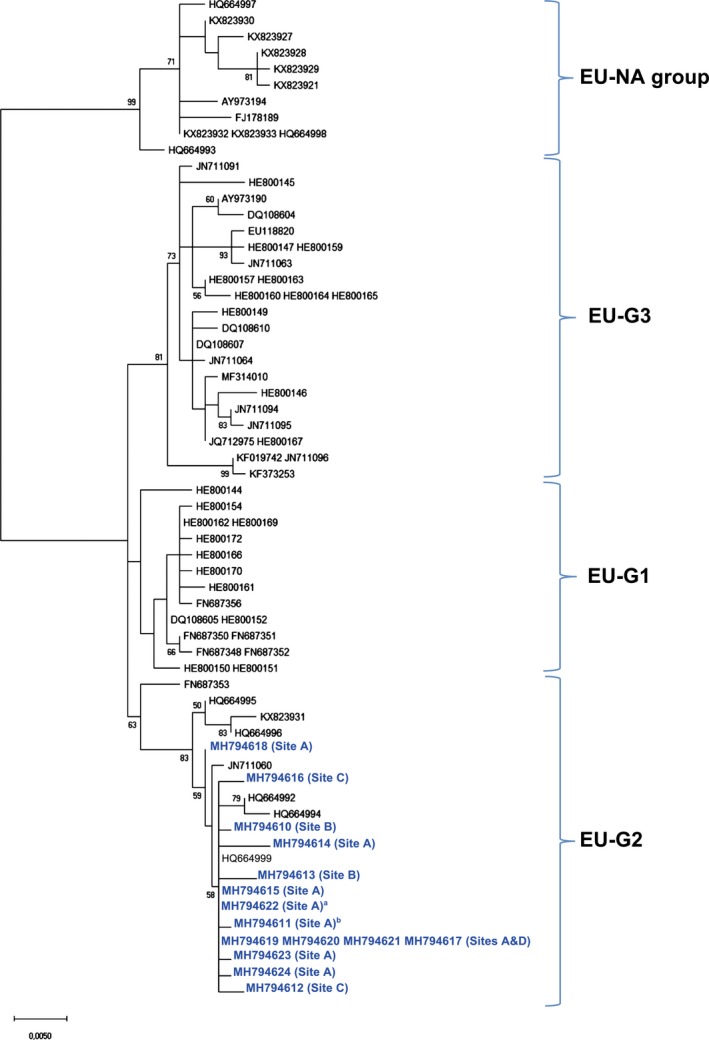
Molecular phylogenetic analysis by the maximum‐likelihood method; K2 + G model, MEGA X (Kumar, Stecher, Li, Knyaz, & Tamura, 2018). The tree with the highest log‐likelihood (−2173,50) is shown. It is based on 78 nucleotide sequences (62 unique) and 834 nucleotides. The sequences obtained in the current study are shown in blue. The percentage of trees in which the associated taxa clustered together is shown next to the branches, unless <50%. Scale bar: substitutions per site. The major groups (clades) previously identified are indicated. ^a^Sequence from escaped farmed salmon. ^b^Shorter sequence added by parsimony (99.8% identity, 73.5% coverage) [Colour figure can be viewed at http://www.wileyonlinelibrary.com]

We obtained partial sequences of fusion protein gene (segment 5) from 7 fish. The sequences were closely and grouped with members of clade 5M (Plarre et al., 2012), showing highest identity (99.1%–99.6%) with sequences found in farmed Atlantic salmon from the far north of Norway (data not shown). It has been suggested that either a Q266L substitution or an insertion in sequence near the cleavage site of the fusion protein gene is among the virulence markers of ISAV (Markussen et al., 2008). As expected in HPR0 virus, none of the present sequences of segment 5 had these markers.

In summary, we have investigated the occurrence and the genotypes of ISAV in wild salmon from northern Norway and revealed only ISAV‐HPR0 infections. The prevalence showed no apparent relationship to fish farming. All the HE‐gene sequences of ISAV‐HPR0 obtained in the current study were closely related and belonged to the EU‐G2 genogroup, which suggests that this genogroup is dominating in wild Atlantic salmon in northern Norway. These findings highlighted the need for more studies about the prevalence and phylogeographical structure of ISAV in wild Atlantic salmon populations.

## CONFLICT OF INTEREST

The authors have no conflict of interest to declare.
