# Supplementary material for: Prevalence and genotypes of infectious salmon anaemia virus (ISAV) in returning wild Atlantic salmon (Salmo salar L.) in northern Norway
Source: J Fish Dis. 2019 Jun 13;42(8):1217–21. doi: 10.1111/jfd.13021 (PMC6851747; doi:10.1111/jfd.13021)
Supplement: Supplementary file 1 [file JFD-42-1217-s001.docx]

**
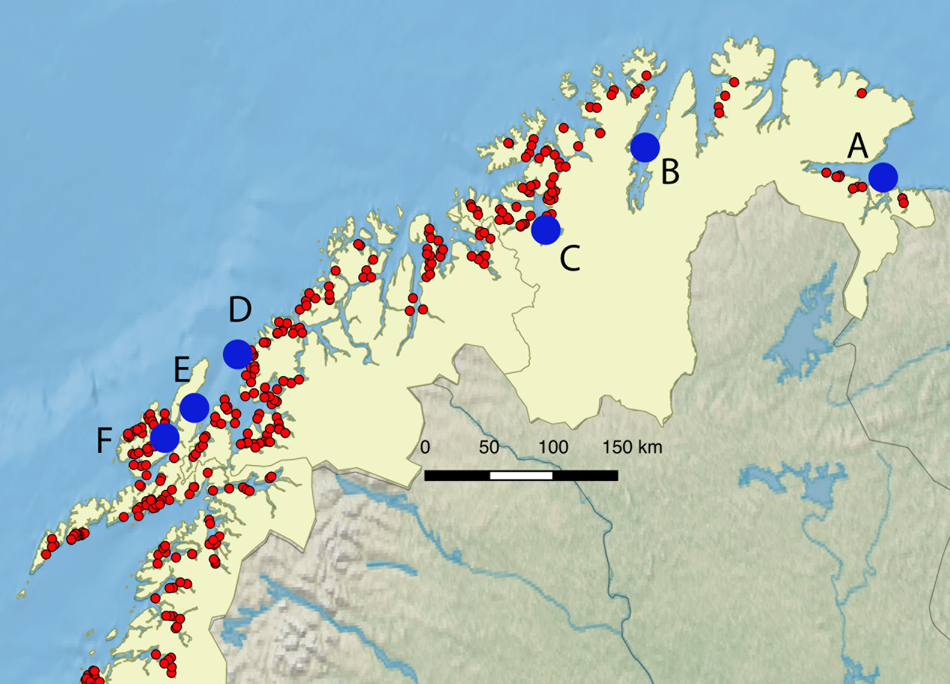
**

**Supplementary figure 1**: Map showing the location of salmon catching sites (blue circle) and fish farms (red circle).
