# Supplementary material for: Prevalence and genotypes of infectious salmon anaemia virus (ISAV) in returning wild Atlantic salmon (Salmo salar L.) in northern Norway
Source: J Fish Dis. 2019 Jun 13;42(8):1217–21. doi: 10.1111/jfd.13021 (PMC6851747; doi:10.1111/jfd.13021)
Supplement: Supplementary file 2 [file JFD-42-1217-s002.docx]

**Supplementary table 1:** The primers used in the amplification and sequence of segments 5 and 6.

| Segment | Name | Sequence (5'–3') | Reference |
| --- | --- | --- | --- |
|  |  |  |  |
| 6 | HansF3 | CATCCCAACTTCGATGACACTGG | (Vike et al., 2009) |
| 6 | ISAV HPR Rev | AGACAGGTTCGATGGTGGAA | (Kibenge et al., 2009) |
|  |  |  |  |
| 5 | S5-F1 | AGTTAAAGATGGCTTTTCTAACAATT | (Vike et al., 2009) |
| 5 | S5-F10 | ACCAAACAAAAGTTAAAGATGG | (Vike et al., 2009) |
| 5 | S5-R3 | TTCTAAATTATCCAATAAAGGTCCTG | (Vike et al., 2009) |
| 5 | S5-R10 | CAAAATATAAGTTATGTACAG | (Vike et al., 2009) |
